# Future Prospects for Using Clinical Phenotypes in Tuberculosis Precision Medicine—An Approach for Clinical Management

**DOI:** 10.1093/cid/ciaf663

**Published:** 2026-01-15

**Authors:** Katarina Niward, Clara Braian, Gustav Svärdhagen, Daniel Augustinsson, Isabelle Öhrnberg, Rovina Ruslami, Stellah Mpagama, Elin M Svensson, Judith Bruchfeld, Jan-Willem Alffenaar, Thomas Schön

**Affiliations:** Department of Infectious Diseases, Region Östergötland, Linköping University, Linköping, Sweden; Department of Biomedical and Clinical Sciences, Division of Inflammation and Infection, Linköping University, Linköping, Sweden; Department of Biomedical and Clinical Sciences, Division of Inflammation and Infection, Linköping University, Linköping, Sweden; Department of Biomedical and Clinical Sciences, Division of Inflammation and Infection, Linköping University, Linköping, Sweden; Department of Infectious Diseases, Region Östergötland, Linköping University, Linköping, Sweden; Department of Biomedical and Clinical Sciences, Division of Inflammation and Infection, Linköping University, Linköping, Sweden; Department of Infectious Diseases, Region Östergötland, Linköping University, Linköping, Sweden; Department of Biomedical and Clinical Sciences, Division of Inflammation and Infection, Linköping University, Linköping, Sweden; Department of Biomedical Sciences, Faculty of Medicine, Universitas Padjadjaran, Bandung, Indonesia; Department of Infectious Diseases, Kibong’oto Infectious Diseases Hospital, Kilimanjaro, Tanzania; Nelson Mandela African Institution of Science and Technology, Arusha, Tanzania; Department of Pharmacy, Uppsala University, Uppsala, Sweden; Department of Pharmacy, Pharmacology and Toxicology, Radboud University Medical Center, Nijmegen, The Netherlands; Department of Infectious Diseases, Karolinska University Hospital, Stockholm, Sweden; Department of Medicine Solna, Division of Infectious Diseases, Karolinska Institutet, Stockholm, Sweden; Sydney Institute for Infectious Diseases, The University of Sydney, Sydney, New South Wales, Australia; School of Pharmacy, The University of Sydney Faculty of Medicine and Health, Sydney, New South Wales, Australia; Department of Pharmacy, Westmead Hospital, Sydney, New South Wales, Australia; Department of Infectious Diseases, Region Östergötland, Linköping University, Linköping, Sweden; Department of Biomedical and Clinical Sciences, Division of Inflammation and Infection, Linköping University, Linköping, Sweden; Department of Infectious Diseases, Kalmar County Hospital, Linköping University, Kalmar, Sweden

**Keywords:** tuberculosis, precision medicine, treatment monitoring, stratified treatment, disease severity

## Abstract

Developing shorter treatment regimens for tuberculosis requires careful characterization of the clinical phenotype, which is defined by patient characteristics, radiological extent of disease, mycobacterial burden, drug susceptibility, and host response. Advances in ’omics and model-informed precision dosing, as well as integrated algorithms using artificial intelligence, need to be adapted and validated in clinical trials to improve classification of patients for stratified treatment. When treatment is initiated based on the clinical phenotype, monitoring of treatment response can be improved by quantification of bacterial load, transcriptomic and epigenetic biosignatures for sputum-free monitoring, and assessing disease burden by radiological and symptom scoring tools. Many of these tools are suitable for high-endemic settings. Such integrated monitoring allows prompt drug adjustments for rapid reduction in bacterial load, which prevents development of drug resistance and achieves relapse-free cure even with shorter treatment.

## Teaser

This review explores current and future tools for defining and managing clinical tuberculosis phenotypes from a clinical perspective. Treatment monitoring using bacterial quantification and ’omics with model-informed precision dosing may reduce relapse and improve patient selection for stratified shorter regimens.

Once tuberculosis (TB) disease is diagnosed, treatment length is still 4–6 months. In clinical trials exploring shorter treatments of drug-susceptible (DS) and multidrug-resistant (MDR) TB [1–3], there is no consensus or standardized strategies on how to classify the extent of TB disease before treatment. Currently, crude characteristics, such as lung cavities on chest radiology and bacillary burden measured by smear microscopy, are used for pulmonary TB (PTB). Biomarkers, such as transcriptomic and proteomic signatures in peripheral blood [4], have recently emerged but are rarely considered for characterizing initial disease severity. Additionally, the translation of such novel strategies into clinical practice in both high- and low-endemic TB areas is slow.

After commencement of TB treatment, biomarkers predictive of relapse-free cure based on reduction in bacterial burden and radiological changes in conjunction with therapeutic drug monitoring (TDM) using early model-informed precision dosing (MIPD) can support decisions on treatment [5]. New approaches for early TDM are explored, using MIPD and collection of noninvasive samples, such as urine and saliva, to facilitate testing for all patients with TB.

Ideally, TB treatment is based on individual characteristics and initial disease severity, so-called stratified treatment, which is then modified by early treatment monitoring to optimize drug exposure with the goal of selecting the shortest treatment duration and most efficient treatment regimen. Current TB treatment mainly relies on chemotherapy by antibiotics but may, in the future, also include host-directed therapies based on the immune phenotype defined within the clinical phenotype. This perspective outlines the pathway towards implementation of a characterization of the clinical phenotype and monitoring of treatment response to improve clinical management in TB.

## CLINICAL PHENOTYPE

**Figure 1.**
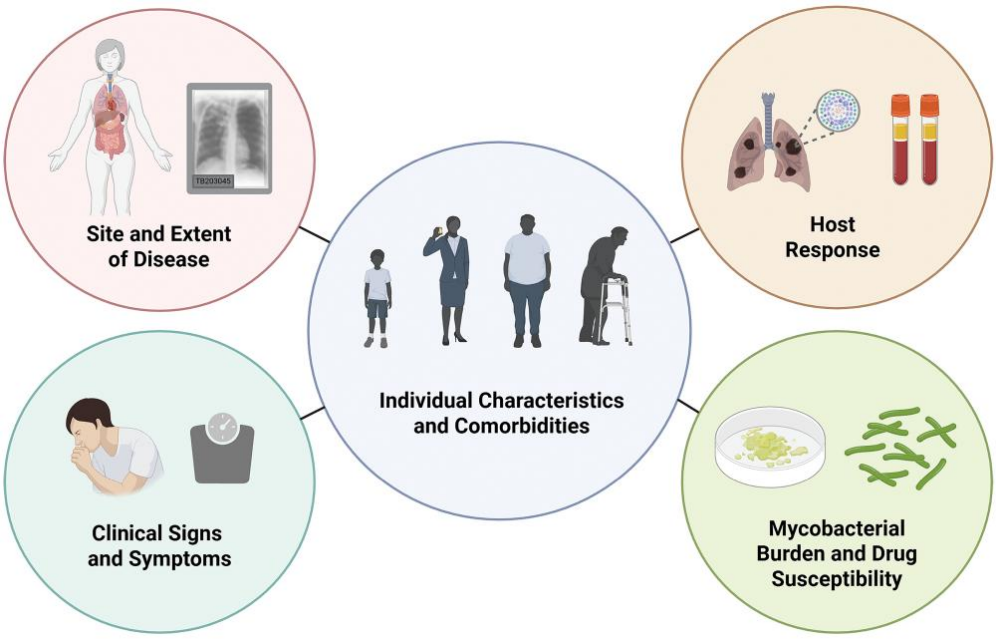
Overview of the clinical TB phenotype. Currently available methods can define the clinical phenotype, based on individual characteristics and comorbidities (center circle) combined with TB disease–related factors, such as the site and extent of TB disease (including radiological features like cavities and miliary patterns in PTB), clinical signs and symptoms, assessment of mycobacterial burden and drug susceptibility, as well as markers and signs of host response. Abbreviations: PTB, pulmonary tuberculosis; TB, tuberculosis.

**Figure 2.**
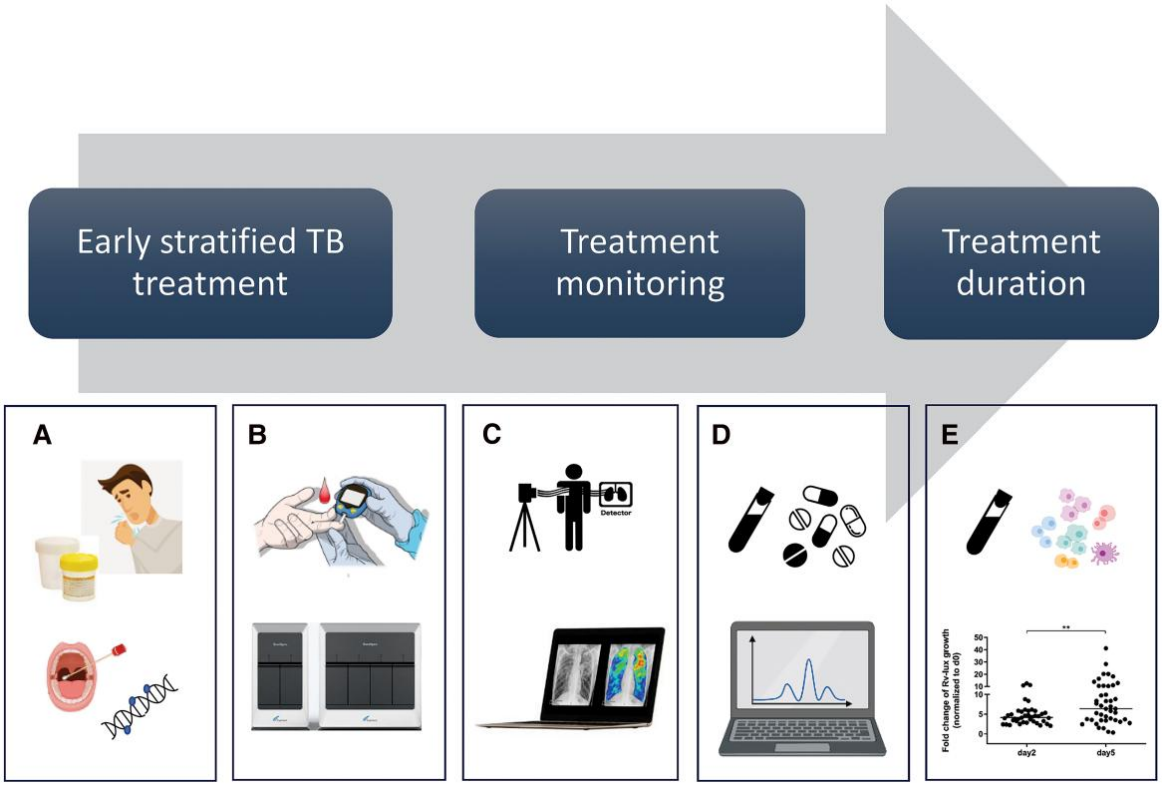
The 3 main stages in clinical decision-making for TB treatment. *A, B,* Clinical TB phenotype defined by current measures of disease activity developed into point-of-care PCR tests based on mouth swab for host epigenetic biosignature, finger prick for host transcriptome expression, quantification of RNA expression from live mycobacteria, and genotypic drug susceptibility testing directly on the sample for early stratified treatment. *C*, In pulmonary TB, computerized chest X-ray may contribute to better staging of the phenotype regarding extent of disease. *D*, Early optimized dosing by model-informed precision dosing can rapidly ensure individualized adequate drug exposure while minimizing side effects. *E*, In the future, surrogate markers of host immune response against *Mycobacterium tuberculosis* such as the mycobacterial growth inhibition assay, can be added to tailor treatment where patients with weak ability to kill mycobacteria may need increased drug exposure and benefit the most from early model-informed precision dosing. *A–E,* If treatment is optimized with precision medicine including prediction of relapse-free cure, treatment can be personalized and most likely significantly shortened to avoid today's over- and undertreatment following “one-size-fits-all” regimens. Abbreviations: PCR, polymerase chain reaction; TB, tuberculosis.

From the clinical perspective, the TB phenotype includes disease severity and is based on the individual's characteristics and comorbidities, site and extent of TB disease, clinical signs and symptoms, mycobacterial burden, drug susceptibility, and host response, reflecting components from both the host and pathogen (Figure 1). The tools available need adaptation depending on the resources at hand (Table 1). When the clinical TB phenotype has been characterized, this can be used to guide the 3 main clinical decision-making steps during TB treatment (Figure 2), as follows: (1) an early stratified approach to design the TB treatment based on characterizing the clinical phenotype, (2) early treatment monitoring to optimize drug-dosing regimen, and (3) determining the treatment duration.

**Table 1.** Current and Future Aspects on Precision Medicine in Tuberculosis Disease Towards a Stratified Approach to Treatment

	Stratified TB treatment based on:		Treatment monitoring based on:		Treatment duration based on:
	A. Patient characteristics and comorbidities; B. Site/extent of disease; C. Clinical signs/symptoms	D. Mycobacterial burden and Mtb DST; E. Drug exposure; F. Host response	A. Adherence; B. Response in clinical signs/symptoms and in extent of disease	C. Mycobacterial burden and replication rate; D. Drug exposure; E. Host response	Clinical phenotype Adherence Future biomarkers of relapse-free cure
**Basic tools**	A. Gender, age, HIV, hepatitis, diabetes mellitus, smoking B. PTB-EPTB, CXR C. Degree of symptoms, TBscore, weight, BMI	D. Point-of-care tests: • PCR/NAAT • MTB/RIF • Smear grade E. NA F. IGRA for T-cell anergy Point-of-care tests: • eSR, CRP	A. Patient-centered care • VOT • Smart pill box • Modified DOT B. Relief of symptoms, TBscore, weight, BMI, CXR	C. Sputum smear conversion D. NA E. Point-of-care tests: • eSR, CRP	**STANDARD TREATMENT OR EXTENDED TREATMENT**
**Advanced tools**	A. NA B. CT C. NA	D. TTP in sputum, NGS DST, DST by culture E. TDM, NAT2 test F. Point-of-care tests: • Proteomic signatures • Transcriptomic signatures	A. Person-centered care B. CT	C. Sputum culture conversion, TTP, TB-MBLA D. TDM E. Point-of-care tests: • Proteomic signatures • Transcriptomic signatures	**STANDARD TREATMENT OR EXTENDED TREATMENT**
**Future tools**	A. Automatic AI-based scoring from medical records B. Point-of-care tests: • CXR CAD_severity_ C. NA A–F. AI-scoring tool based on machine learning	D. Point-of-care tests: • TB-MBLA • NGS DST E. TDM by urine or saliva; early MIPD F. MGIA Point-of-care tests: • Integrated–’omics	A. Person-centerd care B. PET-CT Point-of care tests: • CXR CAD_monitor_ A–E. AI algorithm to predict relapse-free cure	C. Point-of care tests: • TB-MBLA • RS ratio D. TDM by urine or saliva MIPD by treatment response E. MGIA Point-of-care tests: • Integrated–’omics	**TOWARDS SHORTER TREATMENT WITH RELAPSE-FREE CURE**

Host response includes both markers of Mtb infection such as ’omics signatures but also stimulated immune responses such as IGRA and MGIA.

Abbreviations: AI, artificial intelligence; BMI, body mass index; CAD, computer-aided detection; CRP, C-reactive protein; CT, computed tomography; CXR, chest X-ray; DOT, directly observed therapy; DST, drug-susceptibility testing; EPTB, extrapulmonary tuberculosis; eSR, erythrocyte sedimentation rate; HIV, human immunodeficiency virus; IGRA, interferon-gamma release assay; MBLA, molecular bacterial load assay; MGIA, mycobacterial growth inhibition assay; MIPD, model-informed precision dosing; Mtb, *Mycobacterium tuberculosis*; NA, not applicable; NAT2, N-acetyltransferase 2; NGS, next-generation sequencing; PCR/NAAT, polymerase chain reaction/nucleic acid amplification test; PET-CT, positron emission tomography–computed tomography; PTB, pulmonary tuberculosis; RS ratio, the ribosomal RNA (rRNA) synthesis ratio of Mtb; TB, tuberculosis; TB-MBLA, tuberculosis molecular bacterial load assay; TDM, therapeutic drug monitoring; TTP, time to positivity; VOT, video-observed therapy.

## INITIATION OF STRATIFIED TB TREATMENT BASED ON THE CLINICAL PHENOTYPE

### Rationale and Current Perspectives for Characterizing Clinical Phenotypes and Disease Severity in TB

Recent evidence suggests that TB represents a disease spectrum where incipient and subclinical asymptomatic TB have been introduced in-between TB infection and TB disease [6]. The understanding of TB disease itself as a heterogeneous condition—ranging from mild to severe forms—has largely been overlooked [6]. The heterogeneity and lack of standardization of the clinical TB phenotype affects interpretation and comparability of clinical studies (Table 2). Among larger randomized clinical trials exploring shortening of TB treatment, pulmonary cavities on chest X-ray (CXR), sputum smear grade, and body mass index (BMI) at baseline are included in most, but not all, studies and for other biomarkers there is a heterogenous selection and inconsistency in methodological approaches (Table 2) [1–3, 7, 8] and there are still considerable research gaps for new biomarkers (Textbox). An easy-to-treat PTB disease phenotype has been suggested based on a pooled retrospective analysis of clinical trials (n = 3405), defined by a low smear grade and the absence of cavitary disease, whereas a high smear grade and human immunodeficiency virus (HIV) coinfection are associated with a hard-to-treat phenotype [9]. From the Tuberculosis Trials Consortium Study 31 on treatment shortening by rifapentine-moxifloxacin, a simple classification of a moderately harder-to-treat group in-between easy-to-treat and hard-to-treat has been suggested by introducing GeneXpert MTB/RIF cycle threshold values less than 18 and extent of disease > 50% on CXR [10]. Prospective studies such as SPECTRA-TB and PRISM-TB (NCT06441006) will focus on matching shorter TB treatment regimens to the easy-to-treat and hard-to-treat phenotypes.

**Table 2.** Baseline Characteristics Related to Clinical Phenotypes of Patients With Pulmonary Tuberculosis Reported in Recent Randomized Controlled Treatment-Shortening Trials

	EndTB	Truncate TB	TB-PRACTECAL	Study 31/A5349	ReMox
Publication year	2025	2023	2022	2021	2014
Total patients, n	699	674	152	2343	1076
HIV, n (%)	98 (14)	ND	41 (27)	194(8)	110 (7)
Diabetes, n (%)	105 (15)	ND	ND	ND	ND
BMI, median, kg/m^2^	20.9 (IQR: 18.0–22.8)	19.0 (r: 12–33)	19.9 (IQR: 17.3–22.3)	18.9 (r: 12.8–45.2)	ND
Cavity on CXR, n (%)	399 (57.1)	363 (54)	95 (62.5)	1718 (63)	1092 (71)
Cavity >4 cm on CXR, n (%)	154 (22.1)	11 (2)	ND	929 (40)	ND
Smear grade negative, n (%)	131 (18.7)	191 (28)	64 (35.5)	88 (3.8)	0
Smear grade 3+, n (%)	250 (35.8)	72 (10.7)	ND	621 (26.6)	ND
Xpert MTB/RIF (high), n (%)	ND	122 (19)	ND	ND	ND
TTP d, n (%)	ND	ND	ND	ND	<5, n = 722 (47)
Relapse risk^a^ “low”, n (%)	ND	193 (29)	ND	ND	ND

Abbreviations: BMI, body mass index; CXR, chest X-ray; DM, diabetes mellitus; HIV, human immunodeficiency virus; ND, no data available in publication or appendices; r, range; TB, tuberculosis; TTP, time to positive culture in BACTEC 960 MGIT.

^a^Low relapse risk: negative smear, cavity on CXR not more than 4 cm. Intermediate relapse risk: positive smear of 2+ or lower, cavity on CXR <4 cm. High relapse risk: smear 3+ or cavity >4 cm or both.

**Textbox.** Research Gaps

A prognostic biomarker or biosignature for relapse-free cure measurable within the first weeks after diagnosis to support treatment shortening
A prognostic biomarker for post-TB cardiovascular, cancer, and respiratory disease
A real-time biomarker or biosignature indicating an early, favorable treatment response
Model-informed precision dosing strategies for extrapulmonary TB
Machine-learning models for evaluating CXR and PET-CT that correlate with relapse-free cure
A method for quantifying viable bacterial load that gives results within 1 day
Development of standardized biomarkers and biosignatures for defining the clinical TB phenotype including disease severity and evaluated to include the full scale of immune phenotypes
Rapid genotypic-AST directly from the sample with similar sensitivity as culture (tNGS)
How to use host-directed therapy in relation to results from ’omics methods and mycobacterial growth inhibition assays in patients with TB with poor cell-mediated immunity and poor response to treatment
AI-driven models based on the clinical phenotype to decide upon treatment regimen and treatment duration

Abbreviations: AI, artificial intelligence; AST, antimicrobial susceptibility testing; CXR, chest X-ray; PET-CT, positron emission tomography–computed tomography; TB, tuberculosis; tNGS, targeted next-generation sequencing.

### Considerations on Comorbidities in TB

Immunosuppression, such as HIV, diabetes mellitus (DM), and undernutrition, affects cell-mediated immune responses, and has an impact on TB treatment outcome [6]. A CD4 count of less than 350 cells/mm^3^ has been associated with signs of wasting and low BMI even in HIV-negative patients with TB [11], indicating increased disease severity. In a meta-analysis [12], DM was a significant risk factor for unsuccessful treatment outcome of drug-resistant TB (odds ratio [OR], 1.56; 95% CI, 1.24–19.6). Several studies indicate that a BMI lower than 17–18.5 kg/m^2^ is linked to increased TB disease severity and a poor prognosis, with a pooled analysis showing a hazard ratio (HR) of 1.4 per 5-kg/m^2^ decrease (95% CI, 1.1–1.7) for an unfavorable treatment outcome [9].

### Radiological Grading of the Extent of TB Disease

A recent meta-analysis showed that bilateral disease and presence of cavities were associated with an unfavorable treatment outcome (HR, 1.7 [95% CI, 1.2–2.4], and HR, 1.2 [95% CI, 1.0–1.5], respectively) [9]. Furthermore, smear-positive individuals with cavitary lesions at baseline have a significantly higher risk of mortality and failure/recurrence (OR, 1.5 [95% CI, 1.1–2.1], and 2.2 [95% CI, 1.5–3.3], respectively) [13]. Disease extent on CXR of 50% or greater and bilateral disease on CXR at baseline have also been associated with unfavorable outcomes (HR, 2.02; 95% CI, 1.07–3.82) [10].

Both in high- and low-resource settings, artificial intelligence–based CXR and computer-aided detection (CAD) CXR are comparable to radiologists’ assessments in identifying TB by screening [14]. In a small study, we showed that, in addition to identifying TB-related pathology, CAD CXR algorithms are also promising for disease stratification as they are associated with markers of disease severity, such as smear microscopy, time to positive culture (TTP), C-reactive protein (CRP), and BMI [15]. In some CAD software—for example, Qure.ai—radiographic features such as fibrosis, pleural effusion, cavity size, and consolidation are scored separately, which may be used to optimize radiological definition of the clinical phenotype [15]. Chest X-ray is the most widely available and used for grading of TB disease, but in high-resource settings both computed tomography (CT) and positron emission tomography (PET)–CT have been used for TB disease stratification [6, 16].

### Clinical Scoring Tools Based on Symptoms and Signs

For disease stratification, the Bandim TBscore [17] correlates with outcome, where a score > 8 significantly correlated to a 21% mortality rate (45/218) over 8 months, compared with 11% (55/480) for those with a score < 8. The Bandim TBscore also correlated with other severity markers of TB disease, such as CXR grading and biomarkers [18].

### Mycobacterial Burden

Patients with grade 3+ smear-positive TB at baseline have a higher risk of unfavorable outcomes compared with patients with smear-negative or grade 1 smear-positive TB in a patient-level pooled analysis of treatment-shortening regimens for DS-PTB (adjusted HR [aHR], 1.4; 95% CI, 1.1–1.9) [9]. Several rapid diagnostic platforms are recommended by the World Health Organization, such as GeneXpert MTB/RIF, which show a strong correlation with smear status (*r* = 0.78). Every 3-step cycle of threshold decrease from a baseline cutoff of 18 was linked to an HR of 1.69 (95% CI, 1.1–2.6) for unfavorable outcome in DS-TB [10]. Rapid diagnostic platforms are considered useful tools to assess initial mycobacterial load, due to accessibility in low-resource settings.

The TB–molecular bacterial load assay (MBLA) measures ribosomal RNA (rRNA) from viable bacteria and was recently shown to have a high pretreatment diagnostic sensitivity (99%) and specificity (90%) compared with GeneXpert [19] and TTP. However, TTP is less available and may take up to 4 weeks until results, making it unsuitable for initial disease stratification.

### Antimicrobial Susceptibility Testing

Early antimicrobial susceptibility testing (AST) results are important for deciding on an adequate treatment strategy, including increased dosing in low-level drug resistance such as to isoniazid by the *inhA* c-14 mutation [20]. Current whole-genome sequencing (WGS) techniques require mycobacterial DNA from a positive culture, which is time-consuming. Recent advances in targeted next-generation sequencing (tNGS) techniques, such as Deeplex Myc-TB and AmPORE TB, enable sampling directly from sputum, are becoming adapted to high-endemic areas and can report on major drug-resistance genes within a few days [21]. A recent meta-analysis reported an overall sensitivity and specificity of 94% and 98%, respectively, compared with WGS-based AST or phenotypic AST when examining 29 drug-susceptibility studies using tNGS [21].

### Host Immune Response Biomarkers

The host immune response includes both biomarkers of *Mycobacterium tuberculosis* (Mtb) infection such as ’omics signatures but also host immune responses by stimulation of Mtb and its antigens. Of the host immune response markers used in clinical routine, CRP has been associated with severity of TB, with higher levels related to > 50% lung involvement on CXR [22]. T-cell anergy, defined by depressed interferon-gamma (IFN-γ) responses measured by the mitogen-control in interferon-gamma release assay (IGRA) tests, was linked to failure of sputum conversion at 2 months (OR, 2.49; 95% CI, 1.05–5.90; n = 212) [23]. Another promising development has been from transcriptomics where RNA signatures in peripheral blood have been linked to TB disease and to the prognosis in clinical multicenter studies [24, 25].

## EARLY TREATMENT MONITORING AND OPTIMIZATION

The dynamics of surrogate markers of TB disease severity following the initial treatment response is important for clinical decision making (Table 1). Individuals may initially experience extensive and severe TB disease but still respond rapidly to treatment.

### Monitoring Clinical Scoring, Mycobacterial Load, and Replication Rate

A less than a 25% decline in Bandim TBscore version II between treatment initiation and 2-week follow-up was associated with increased mortality and higher failure rate [26]. Smear microscopy and DNA-based methods are not useful for follow-up of bacterial killing as they detect both dead and live bacilli [19]. Quantifying mycobacterial rRNA using MBLA needs adaptation to become suitable for routine laboratories. However, in a study of 129 patients with Xpert positive TB, MBLA decreased at the same rate as TTP during the first 2 months of treatment and correlated with stable culture conversion in contrast to smear microscopy and Xpert, which were found to be unsuitable for treatment monitoring [19]. Additionally, the turnaround time for MBLA is 1–2 days compared with several weeks for TTP, making MBLA suitable as an early marker of treatment response [4, 19]. The Mtb rRNA synthesis (RS) ratio quantifies the bacterial replication rate by comparing precursor rRNA with the amount of stable 23S rRNA in contrast to traditional assays measuring bacterial load, such as TTP or MBLA. The RS ratio could be useful to measure drug activity in sputum during treatment, which may be a useful tool for treatment shortening [27].

### Dose Optimization by Therapeutic Drug Monitoring

Subtherapeutic drug concentrations during TB treatment are common, and a meta-analysis showed increased risk of a poor outcome for patients with low pyrazinamide concentrations (8 studies, n = 2727; relative risk [RR], 1.73; 95% CI, 1.10–2.72) and with low rifampicin concentrations (13 studies; n = 2753; RR, 1.40; 95% CI, .91–2.16) [5]. Pharmacokinetic/pharmacodynamic (PK/PD) targets for TB treatment predictive of successful treatment are essential for TDM [28]. Reasons for PK variability and suboptimal drug exposure are multifactorial, including factors such as drug formulation, body composition, malabsorption, DM, HIV, and the N-acetyltransferase 2 (NAT2) phenotype —where rapid acetylators have an increased risk of low isoniazid exposure [5, 12, 29]. Early TDM is particularly important for immunocompromised patients and those with severe disease where the risk of acquired drug resistance is high. As traditional TDM relies on drug concentrations at steady state, which can take weeks, faster approaches have been developed. Model-informed precision dosing integrates population PK models with individual drug concentrations and patients’ characteristics to predict optimal dosing even before steady state [30, 31]. Dosing can be adjusted based on these predictions, aiming to improve treatment outcomes by avoiding toxicity and ensuring drug efficacy [30]. Several applications have been developed to assist with dosage adjustments of first- and second-line TB drugs (eg, Tucuxi, MwPharm, and InsightRX) [30].

Noninvasive methods using saliva or urine for the measurement of drug exposure provide simple point-of-care tests with semiquantitative results of drug concentrations, which can help detect patients with low or high drug exposure at the community level [32], while TDM using more advanced liquid chromatography–mass spectrometry can be offered at reference centers for selected cases [33].

### Host Biomarkers as Monitoring Tools

Among the ’omics methods, few clinical studies include disease severity and longitudinal follow-up, which currently prevents their routine use in defining the clinical phenotype. Host-response–mediated mRNA expression in peripheral blood samples using real-time–polymerase chain reaction (RT-PCR) does not require sputum sampling. The Xpert-MTB-Host Response is based on a 3-gene signature [34] and was shown to correlate with the extent of lung pathology at treatment start in a South African cohort of individuals with TB disease [25]. Further, the 3-gene score remained higher in those with persistent lung inflammation during and at the end of treatment, compared with those with radiological improvement [25]. Xpert-MTB-HR has been developed for the GeneXpert cassette-based system, making it suitable for point-of-care settings. RISK6 is a 6-gene mRNA signature, which decreased during TB treatment, significantly predicting treatment outcome defined as negative culture at 6 months after treatment (area under the curve [AUC], 77.1; 95% CI, 52.9–100; n = 85) [24]. The TB-Host Immune Response Assay (TB HIRA) test is a commercial assay being developed as a point-of-care test based on RISK6 [24].

While RNA signatures are the most widely studied, metabolomics and proteomics have also been investigated for treatment monitoring. In a meta-analysis of treatment-monitoring biomarkers in blood for TB [28], CRP, interleukin (IL)-6, Interferon gamma-induced protein 10 (IP-10, and tumor necrosis factor alpha (TNF-α) most strongly correlated with treatment response at 8 weeks of treatment compared with baseline when examining 77 different studies. A decrease of less than 55% of CRP at 2 weeks after treatment initiation has been shown to correlate with death or hospitalization [35].

Epigenetic modifications of DNA control inflammatory processes by regulating transcription of genes [36]. Recently, such epigenetic biosignatures were shown to change significantly during TB treatment, indicating its prognostic potential [37].

### Ex Vivo Functional Measurement of Host Immune Response

Mycobacterial growth inhibition assays using peripheral blood mononuclear cells (PBMCs) from individuals with TB disease can be used to assess host immune responses to Mtb as a part of the clinical phenotype. A strong anti-mycobacterial control of PBMCs has been found to positively correlate with sputum conversion at 2 months after treatment start [38]. This method also has potential to identify changes in host immune response that may affect treatment outcome [39] as well as potential for monitoring of host-directed therapy in difficult-to-treat cases. Host-directed therapy may include immune boosting and reversal of anergy in patients with poor host responses but also a reduction in immune-induced pathology, such as using corticosteroids to prevent tissue damage in TB meningitis [9].

## TAILORING TREATMENT DURATION BASED ON THE INITIAL RESPONSE AND CLINICAL PHENOTYPE

Within the first 2 weeks of DS-TB treatment most patients show rapid improvement and may have very low contagiousness already after 5 days of treatment, although most guidelines still recommend 2 weeks of isolation [40]. Furthermore, the number and volume of cavitary lesions on CT have been associated with delayed sputum culture conversion [16]. Thus, aspects of the clinical phenotype, such as bacillary burden and extent of CXR findings, in combination with treatment response, may provide possible avenues to assess contagiousness. To ensure a relapse-free cure and achieve the shortest, most efficient treatment regimen, it is crucial to ensure adequate drug exposure alongside clinical, radiological, and microbiological improvement.

The relapse rate for pulmonary DS-TB is, at most, 5%, with two-thirds of relapses occurring within 6 months after treatment [41]. A negative sputum culture at 2 months is the most common tool to predict relapse-free cure in DS-TB [42]. However, its positive-predictive value is only 40%, and it did not correlate with relapse-free cure in clinical trials of treatment shortening using fluoroquinolones, where the relapse rate was 15%–20% [9]. The risk of TB relapse increases to 10% in patients with TB with both positive 2-month sputum culture and pulmonary cavities at baseline [43]. A reduction in lung lesions by more than 50% by the end of an intensive treatment phase has been associated with favorable outcomes. Radiological tools, including CAD CXR, may be used for long-term follow-up but need further study. Other simple tools such as weight gain, included in the TBscore, should be considered as there is a correlation with clinical outcome [44].

Many attempts have been made to find novel biomarkers for treatment monitoring, but few, if any, are predictive of relapse-free cure. A biosignature combining 2 clinical (time to culture positivity and BMI) and 4 immunological (TNF-β, sIL-6R, IL-12p40, and IP-10) parameters at diagnosis, predicted relapse with a 75% sensitivity and 85% specificity [45].

The easy-, moderately, and hard-to-treat phenotypes are being considered as they rely on widely available tools such as CXR, smear microscopy, and Xpert [9, 10], but they need to be prospectively validated in real-world settings as they may be dependent on patient selection and methodological variability (Textbox). Shorter regimens based on a careful definition of the clinical phenotype can improve patient adherence, reduce the risk of drug resistance, and lessen the burden on both healthcare systems and patients [6, 9, 10]. As an example, Truncate-TB is a randomized clinical trial introducing a new strategy with shorter durations of treatment down to 2 months by including linezolid and bedaquiline to the first-line drugs [7]. However, relapse and treatment failure were not uncommon (17% in the intervention arms compared with 3% in standard-of-care) and some patients developed drug resistance, which is a strong argument for including the clinical TB phenotype when selecting patients for shorter treatment regimens. Adding more-advanced TDM and faster drug susceptibility testing is likely to minimize the risk of relapse and development of drug resistance.

## CONCLUSION

The clinical TB phenotype has long been ignored in the design of clinical studies evaluating new drug regimens and shorter treatment strategies. There are still considerable research gaps in defining the clinical phenotype adapted for clinical management with regard to novel biomarkers, surrogate markers of treatment response, and precision dosing (Textbox).

To end TB, we need to move towards a modernized and standardized approach for defining the clinical phenotype beyond smear microscopy, BMI, and CXR and strive to make this available also for high-endemic, low-income settings. This will facilitate treatment shortening by tailoring the regimen according to established clinical phenotypes and dose adjustments based on model-informed precision dosing in combination with point-of-care tests for drug concentration measurements.
